# The homeodomain complement of the ctenophore *Mnemiopsis leidyi *suggests that Ctenophora and Porifera diverged prior to the ParaHoxozoa

**DOI:** 10.1186/2041-9139-1-9

**Published:** 2010-10-04

**Authors:** Joseph F Ryan, Kevin Pang, James C Mullikin, Mark Q Martindale, Andreas D Baxevanis

**Affiliations:** 1 Genome Technology Branch, National Human Genome Research Institute, National Institutes of Health, Bethesda, MD, USA; 2 Kewalo Marine Laboratory, Pacific Bioscience Research Center, University of Hawaii, Honolulu, HI, USA; 3 NIH Intramural Sequencing Center, National Human Genome Research Institute, National Institutes of Health, Bethesda, MD, USA

## Abstract

**Background:**

The much-debated phylogenetic relationships of the five early branching metazoan lineages (Bilateria, Cnidaria, Ctenophora, Placozoa and Porifera) are of fundamental importance in piecing together events that occurred early in animal evolution. Comparisons of gene content between organismal lineages have been identified as a potentially useful methodology for phylogenetic reconstruction. However, these comparisons require complete genomes that, until now, did not exist for the ctenophore lineage. The homeobox superfamily of genes is particularly suited for these kinds of gene content comparisons, since it is large, diverse, and features a highly conserved domain.

**Results:**

We have used a next-generation sequencing approach to generate a high-quality rough draft of the genome of the ctenophore *Mnemiopsis leidyi *and subsequently identified a set of 76 homeobox-containing genes from this draft. We phylogenetically categorized this set into established gene families and classes and then compared this set to the homeodomain repertoire of species from the other four early branching metazoan lineages. We have identified several important classes and subclasses of homeodomains that appear to be absent from *Mnemiopsis *and from the poriferan *Amphimedon queenslandica*. We have also determined that, based on lineage-specific paralog retention and average branch lengths, it is unlikely that these missing classes and subclasses are due to extensive gene loss or unusually high rates of evolution in *Mnemiopsis*.

**Conclusions:**

This paper provides a first glimpse of the first sequenced ctenophore genome. We have characterized the full complement of *Mnemiopsis *homeodomains from this species and have compared them to species from other early branching lineages. Our results suggest that Porifera and Ctenophora were the first two extant lineages to diverge from the rest of animals. Based on this analysis, we also propose a new name - ParaHoxozoa - for the remaining group that includes Placozoa, Cnidaria and Bilateria.

## Background

Ctenophores are a phylum of marine metazoans with uncertain phylogenetic affinity. Their signature morphological features include a set of eight ciliated comb rows that are used for swimming; these are controlled by an aborally located statocyst called the apical sense organ. Most ctenophores have a pair of feeding tentacles that contain specialized adhesive cells called colloblasts. Ctenophores are extremely fragile and difficult to culture and, as such, we know very little about their biology relative to other metazoans [1].

The unique ctenophore body plan has made it difficult to untangle its phylogenetic position in relation to other animal phyla. The earliest comparative classifications by Cuvier allied ctenophores with cnidarians and echinoderms in the Radiata [2]. Later, Leuckart grouped ctenophores with sponges and cnidarians in the Coelenterata [3]. Associations proposed between ctenophores and other taxa include groupings with Platyhelminthes, trochozoans, bilaterians and subsets of sponges and cnidarians (see [4] for a review). Many of the early molecular studies using 18 s ribosomal RNA (rRNA) sequences placed ctenophores sister to Placozoa, Cnidaria and Bilateria (for example, Wainright *et al*., 1993; Smothers *et al*. 1994; Bridge *et al*. 1995; Collins 1998; Kim *et al*. 1999 [5-9]).

The 18 S rRNA placement of ctenophores has recently been challenged using data generated in expressed sequence tag (EST)-based phylogenomic studies. One study re-allies ctenophores with the cnidarians [10], while another has ctenophores branching at the base of the animal tree [11,12]. Yet another study combined morphological, structural and sequence data, leading to the placement of ctenophores in a clade with all other non-bilaterians sister to the Bilateria [13]. These conflicting results could be due to the use of different methods, the inherent incomplete nature of transcript sequencing (in the case of the EST-based studies) or for other reasons. This series of studies have left most investigators waiting for further evidence to tilt the consensus convincingly in one direction or another.

Phylogenomic approaches hold the promise of reconstructing the true tree of life (reviewed in [14]). Thus far, most phylogenomic efforts that have included data from all four of the early branching phyla have been restricted to the aforementioned (and conflicting) EST-based analyses (for example [10-12,15]). Methods based on whole-genome content rather than large concatenated data matrices can provide an independent assessment of current phylogenetic hypotheses and, due to the rarity of events measured, may arguably be more appropriate in this context [14,16]. One such rare genomic change that has previously been employed involves the presence or absence of gene duplications of homeobox genes [17-19].

Homeobox genes encode transcription factor proteins characterized by the presence of a helix-loop-helix DNA-binding domain called the homeodomain [20]. Homeobox genes were present in the last common ancestor of plants, animals and fungi and underwent extensive independent diversification in each of these lineages [21,22]. In animals, the homeobox superfamily has been separated into 11 classes and more than 125 gene families [21,23,24].

Examination of the homeobox complement of species from early-branching metazoan phyla (such as Cnidaria [25,26], Placozoa [27,28] and Porifera [29,30]) has been an especially fertile area of research, one that has been fuelled by the recent availability of full genomic sequence data from several non-bilaterian genomes. The last remaining non-bilaterian phylum lacking a species with a sequenced genome (and, therefore, a completely examined homeobox repertoire) was Ctenophora.

We have used a next-generation sequencing approach to sequence and assemble the ~150 MB of the lobate ctenophore, *Mnemiopsis leidyi*. Here, we present the first whole-genome investigation of the ctenophore homeobox superfamily. Our results expand on previous studies that have utilized degenerate polymerase chain reaction (PCR) approaches to identify ctenophore homeoboxes from the PRD, ANTP and SINE classes [22,31-36]. In addition to these classes, we show that the POU, LIM and TALE classes were also present prior to the divergence of the ctenophores from the rest of Metazoa.

This is also the first study to compare the complete homeobox catalogue of species from all of the non-bilaterian phyla, along with that of the two major bilaterian lineages (Protostomia and Deuterostomia) where complete genomic sequence data is available. As such, this work provides a major missing piece of evidence that is critical to understanding the makeup of the homeodomain superfamily in early metazoan history. With these data in hand, we evaluate the congruency of the homeodomain data with the recently proposed phylogenetic relationships of the early branching phyla.

## Results

### Overview of homeobox genes in *Mnemiopsis*

**Figure 1:**
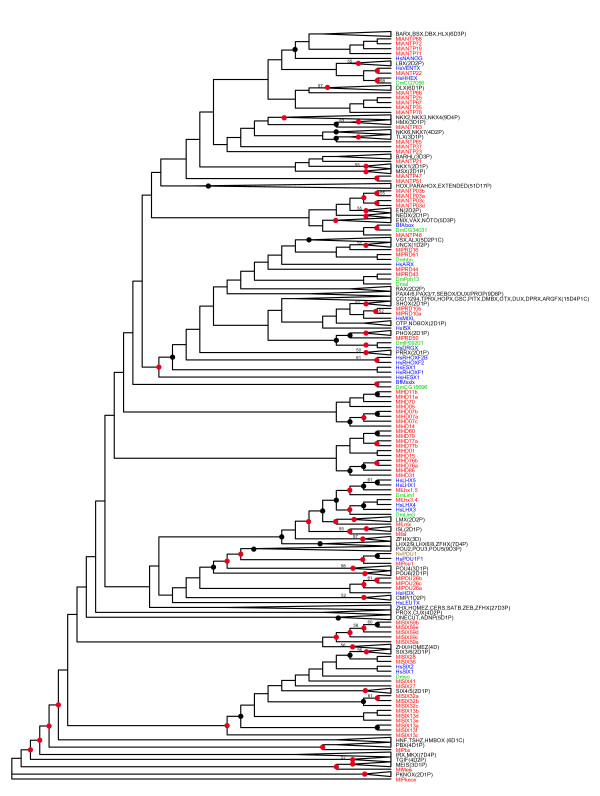
**Homeodomain superfamily tree**. This tree is based on a RaxML tree that included homeodomains from human, *Drosophila*, *Mnemiopsis *and a few related species that serve as place-holders for homeodomains known to be missing from human and *Drosophila *(see Figure 2 legend for species codes). This tree is referred to as a 'superfamily tree' as it includes homeodomains from all classes of the homeodomain superfamily, in contrast to the trees in Figures 2, 3 and 4 that include only homeodomains from individual classes. *Mnemiopsis *sequences are shown in red; human and other deuterostome sequences are shown in blue; *Drosophila *and other protostome sequences are shown in green; and cnidarian sequences are shown in brown. This RaxML tree had a higher likelihood value compared to several other methods and variations of starting trees supplied to RaxML (see methods). Collapsed clades represent clades with no *Mnemiopsis *representative and include a code that indicates how many deuterostome, protostome and cnidarian homeodomains are in that particular clade (for example, 2D4P1C would signify 2 deuterostome, 4 protostome, and 1 cnidarian). ML bootstraps are included for clades with bootstrap values greater than 50. Black dots appear on clades with Bayesian posterior probability values greater than 50 and red dots on clades greater than 90. Rooting of this tree is for display purposes only; branch lengths are presented uniformly, also for display purposes. Actual branch lengths can be viewed by opening the Newick-formatted tree file (Additional File 4), which also includes bootstrap and Bayesian support values, in a tree viewing/editing program such as FigTree [53].

We extracted 76 homeoboxes from the genome of *Mnemiopsis leidyi*. The corresponding homeodomains were aligned to the human and *Drosophila *dataset used in Holland *et al*. 2007 [23] and supplemented with eight amphioxus homeodomains known to be missing from humans. The sequence alignment is available as supplemental material (Additional File 1). We generated nine trees from this alignment using multiple methods (neighbor-joining, maximum likelihood (ML) and Bayesian inference), multiple starting trees and multiple implementations. For example, in the case of ML, we used RaxML [37] and PhyML [38]). In this case, we generated a likelihood value for each tree and then chose the one with the highest likelihood (Figure 1). We subsequently used this tree and secondary domain information, along with the classification scheme in the Homeo Database (HomeoDB) [24], to divide the 76 *Mnemiopsis *homeodomains into the following classes: ANTP (22 homeodomains); PRD (7); TALE (3); POU (4); LIM (4); and SINE (18). Eighteen homeodomains remained unclassified (Table 1).

**Table 1:** *Mnemiopsis *homeobox genes.

Name	Domain	Signature	Intron codons	Accession
**TALE**				
MlPbx	Pbx	insert(NLA) 23	2,47/48	HM444125
MlMeis	MeisA, MeisD	insert(HLT) 23	25/26, 51	HM444091
MlPknox	Pbx	insert(HLG) 23	25/26, 51	HM444122
**POU**				
MlPou1	Pou			HM444110
MlPOU26a	Pou			HM444092
MlPOU26b				HM444093
MlPOU26c				HM444094
**LIM**				
MlIsl	Lim		39	HM444123
MlLhx1.5	Lim		55/56	HM444088
MlLhx3.4	Lim		27	HM444089
MlLmx	Lim		11,55/56	HM444090
**SIX**				
MlSIX13a	Six			HM444111
MlSIX13b	Six			HM444112
MlSIX13c	Six			HM444113
MlSIX13d	Six		12,58	HM444114
MlSIX13e	Six			HM444115
MlSIX13f	Six			HM444130
MlSIX27	Six		17	HM444128
MlSIX28	Six			HM444129
MlSIX32a				HM444116
MlSIX32b	Six			HM444144
MlSIX32c	Six		30	HM444117
MlSIX36	Six	NKL	45	HM444118
MlSIX41	Six		9,45	HM444127
MlSIX59a	Six		57*	HM444131
MlSIX59b	Six		57*	HM444119
MlSIX59c	Six		57*	HM444120
MlSIX59d	Six		57*	HM444121
MlSIX59e	Six		57*	HM444126
**ANTP**				
MlANTP03a		HOXL2		HM444145
MlANTP03b		HOXL2		HM444132
MlANTP03c				HM444134
MlANTP03d				HM444072
MlANTP19		NKL	21/22	HM444073
MlANTP21			10,52	HM444074
MlANTP22			13/14	HM444075
MlANTP23			18	HM444140
MlANTP25				HM444076
MlANTP35			44/45	HM444077
MlANTP37		NKL, HOXL	44-45	HM444078
MlANTP47			46-47	HM444079
MlANTP48		NKL, HOXL2	21-22	HM444080
MlANTP51			36	HM444136
MlANTP63			12/13	HM444137
MlANTP65			12/13,45	HM444081*
MlANTP66			46/47	HM444082*
MlANTP67			17/18,47	HM444083*
MlANTP68		NKL	14/15,44/45	HM444084*
MlANTP71		NKL	9,53	HM444085
MlANTP72		NKL	44/45	HM444086
MlANTP78			39	HM444087
**PRD**				
MlPRD10a		PRD	37	HM444097
MlPRD10b		PRD	24,46/47	HM444098*
MlPRD16	Octapeptide	PRD	46/47	HM444102*
MlPRD43	Octapeptide		46/47	HM444104
MlPRD44		PRD, HOXL2	46/47	HM444105*
MlPRD50	Octapeptide		14/15,46/47	HM444141
MlPRD61	Octapeptide	PRD	12/13,46/47	HM444147
**Unclassified**				
MlHD01				HM444143
MlHD05				HM444146
MlHD07a				HM444139
MlHD07b				HM444095
MlHD07c				HM444096
MlHD11a				HM444133
MlHD11b				HM444099
MlHD14				HM444100
MlHD15				HM444101
MlHD31				HM444103
MlHD60		insert(LP) 33		HM444135
MlHD70				HM444106
MlHD76a				HM444107
MlHD76b				HM444142
MlHD77a				HM444108
MlHD77b				HM444138
MlHD79		insert(N) 22		HM444109
MlHD86				HM444124

The first column (Name) contains the names given to each gene. The second column (Domain) indicates any additional domains detected either in the predicted gene sequence or in close genomic proximity to the homeobox in the same orientation. If NKL, HOXL, HOXL2 or PRD sequence signatures or are present in the translated homeodomain (as defined in [40]), this is noted in the third column. The third column (Signature) also includes the amino acid sequences of atypical insertions if they are present. The format for insertions is the word 'insert' followed by the amino acids that make up the insertion in parentheses and the first codon of the homeobox occupied by the insertion. For those homeoboxes that are interrupted by one or more introns, the fourth column (Intron Codons) lists either the codon that is interrupted (noted by a single number) or the two codons that are separated by an intron (noted by two numbers separated by a forward slash). Commas separate codon positions for genes with multiple introns. The five MlSIX59 homeoboxes are truncated at the 57th codon and occur at the end of their corresponding GENSCAN gene prediction. The last column indicates the GenBank accession of the corresponding nucleotide sequences. An asterisk next to an accession indicates a previously described version of this homeodomain exists. The previously described homeodomains are as follows: MlANTP65 - ACD85820 (Tlx-like), MlANTP66 - ACD85819 (Dlx/NK-like), MlANTP67 - ACD85818 (BarH/BarX-like), MlANTP68 - ACD85817 (Bsh), MlPRD10b - ACD85823 (Prd3), MlPRD16 - ACD85821 (Prd1), MlPRD44 - ACD85822 (Prd2).

Most of these class-level assignments are confirmed by the presence of secondary domains, sequence signatures, and/or class-specific introns (Table 1). To all of these classes (with the exception of the 18 homeodomains that remained unclassified), we added corresponding homeodomain sequence data from the demosponge *Amphimedon queenslandica *[30], the placozoan *Trichoplax adhaerens *[27], the cnidarian *Nematostella vectensis *[26]and the choanoflagellate *Monosiga brevis *[39]: we then performed class-specific phylogenetic analyses. We named *Mnemiopsis *homeodomains that showed a strong affiliation for a particular family accordingly; otherwise, the name of the class is used in conjunction with a preliminary number that was originally assigned to the homeodomain.

### ANTP class NKL subclass

**Figure 2:**
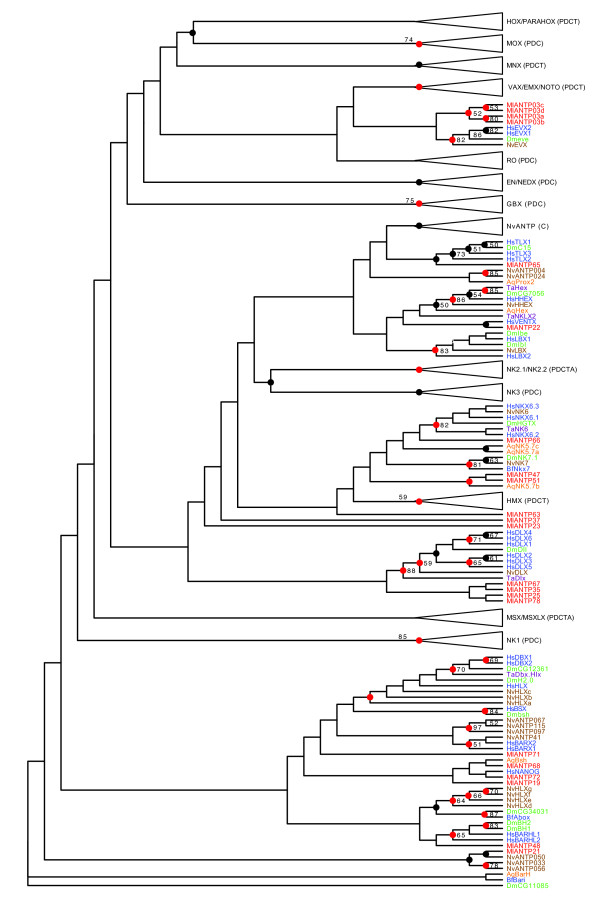
**ANTP tree**. *Mnemiopsis *homeodomains are in red. Arrows represent single genes or clades consisting entirely of *Mnemiopsis *sequences. Collapsed clades (triangles) represent clades with no *Mnemiopsis *representative. These clades have families represented in bold along with the phyla/subkingdom who have sequences represented in the collapsed clade (P = Protostomia; D = Deuterostomia; C = Cnidaria; T = Placozoa; A = Porifera). Support values shown are maximum likelihood (ML) bootstrap greater than 50%. Nodes with posterior probabilities generated by MrBayes greater than 50 are represented by a black dot and those clades with posterior probabilities greater than 90 are represented by a red dot. For visibility, trees were initially formatted in FigTree [53] as cladograms with decreasing ordered nodes and uniform branch lengths. They were later formatted by hand in Adobe Illustrator. Rooting of this tree is for display purposes only; branch lengths are presented uniformly, also for display purposes. Actual branch lengths can be viewed by opening the Newick-formatted tree file (Additional File 4), which also includes bootstrap and Bayesian support values, in a tree viewing/editing program such as FigTree [53]. Homeodomain names are prefixed with two letter species abbreviations as follows: Ml = *Mnemiopsis leidyi; *Aq = *Amphimedon queenslandica *(Porifera/demosponge); Ta = *Trichoplax adhaerens *(Placozoa); Nv = *Nematostella vectensis *(Cnidaria/starlet sea anemone); Dm = *Drosophila melanogaster *(Protostomia/fruitfly); Bf = *Branchiostoma floridae *(Deuterostomia/amphioxus); Hs = *Homo sapiens *(Deuterostomia/human). Other taxa codes either from collapsed clades of this tree or in other trees: Hv = *Hydra vulgaris *(Cnidaria/hydrozoan); Pd = *Platynereis dumerilii *(Protostomia/annelid worm); Am = *Apis mellifera *(Protostomia/honey bee); Ps = *Phascolion strombus *(Protostomia/sipunculan worm); Sm = *Strigamia maritima *(Protostomia/centipede); Mb = *Monosiga brevicollis *(choanoflagellate). *Mnemiopsis *sequences are shown in red; human and other deuterostome sequences are shown in blue; *Drosophila *and other protostome sequences are shown in green; cnidarian sequences are shown in brown; *Trichoplax *sequences are shown in purple; and *Amphimedon *sequences are in orange.

Eighteen of the 22 ANTP homeodomains group are within the NKL subclass. There is only weak support for assigning any of the *Mnemiopsis *NKL homeodomains with particular families but, in some cases, there is consistency between our initial superfamily tree (Figure 1) and our ANTP-specific tree that included the additional *Amphimedon*, *Nematostella*, and *Trichoplax *sequences (Figure 2).

The following groupings are consistent in both trees and have support values over 50 in our best Bayesian tree: (1) MlANTP65 with the Tlx family and (2) MlANTP22 with the Human Ventx gene. MlANPT25, MlANTP35, MlANTP67 and MlANTP78 group with the Dlx family consistently in both trees but, in the full tree, the Dlx clade also includes MlANTP66. Similarly, MlANTP19, MlANTP68, MlANTP71 and MlANTP72 form clades positioned sister to the Barx, Bsx, Dbx and Hlx familes. However, the relationships between *Mnemiopsis *homeodomains is inconsistent between these trees. The other NKL homeodomains identified are MlANTP21, MlANTP23, MlANTP25, MlANTP35, MlANTP37, MlANTP47, MlANTP48, MlANTP51, MlANTP63, MlANTP66, MlANTP67 and MlANTP78.

Consistent with our analysis, a previous study classified MlANTP65 as a Tlx-like homeodomain [36]. The same study also associated MlANTP66 with the Dlx family, MlANTP67 with the Barh family and MlANTP68 with the Bsx family, an observation that was not consistently reproduced in our trees.

Evidence in the form of diagnostic residues can provide additional support to classifications [40]. The following homeodomains all contain the diagnostic residues associated with the NKL subclass ([AKST][DENPS][LAST][Q][V] at positions 41-45): MlANTP19, MlANTP37, MlANTP48, MlANTP68, MlANTP71 and MlANTP72. The only other *Mnemiopsis *homeodomain with the NKL signature is the SINE class homeodomain MlSIX36. (The position of MlSIX36 on the tree in Figure 1 and its upstream SIX domain led to its SINE class designation.) In addition to the NKL signature, the MlANTP37 homeodomain also contains the HOXL signature ([KT][IV]WFQNRR[AMV]K[DEHKLMQWY][KR][KR] at positions 46-58) and the MlANTP48 homeodomains contains the HOXL2 signature (LE[AGKNR]E at positions 16-19) (Table 1).

### ANTP class HOXL-related

Four paralogous *Mnemiopsis *ANTP homeodomains (MlANTP03a, MlANTP03b, MlANTP03c and MlANTP03d) group with the engrailed family in our superfamily tree (Figure 1) and with the Evx family in the ANTP tree (Figure 2). Despite the engrailed family being assigned to the NKL subclass in HomeoDB [24], engrailed has been historically allied with the extended Hox subclass based on synteny [41,42] and phylogeny [43]. Evx is also considered a member of the extended Hox subclass. While it is difficult to pin down the exact relationship of the MlANTP03 homeodomains, it does appear that they are the most likely descendants of the homeodomain that gave rise to the HOXL genes in the lineage leading to Placozoa, Cnidaria and Bilateria. Consistent with this classification, the MlANTP03a and MlANTP03b genes both contain the HOXL2 diagnostic residue signature. There are no clear ParaHox or Hox genes in *Mnemiopsis*.

### PRD class

**Figure 3:**
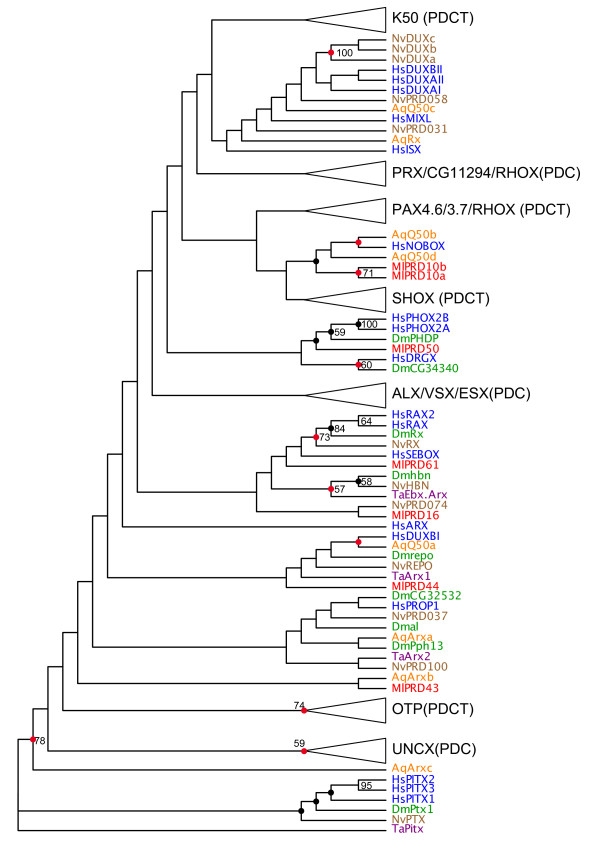
**PRD-HD tree**. See Figure 2 for explanation of tree formatting, color codes and species codes. Rooting of this tree is for display purposes only; branch lengths are presented uniformly, also for display purposes. Actual branch lengths can be viewed by opening the Newick-formatted tree file (Additional File 4), which also includes bootstrap and Bayesian support values, in a tree viewing/editing program such as FigTree [53].

We identified seven PRD class homeodomains in the *Mnemiopsis *genome. The PRD class is divided into three subclasses based on the amino acid residue at position 50: Q50, K50 and S50 [44]. As with most homeodomain studies, these subclasses are not monophyletic in our trees (Figure 3). However, given the extremely low support values at the subfamily level, this may not reflect their true relationship. All three subclasses are clearly present in the genomes of bilaterians, *Nematostella *and *Trichoplax*. Eight of the nine PRD class homeoboxes in *Amphimedon *possess the Q50 residue. The remaining PRD homeodomain is the *Amphimedon *PaxB homeodomain, which is has a degenerate homeodomain [45] and, as such, was not included in our phylogenetic analysis.

Of the seven *Mnemiopsis *PRD class homeodomains, six have a Q at position 50. The exception (MlPRD43) is missing sequence information at that position. (Note: just prior to the submission of this manuscript, a new assembly has revealed the likely 3' end of this homeodomain that includes a Q at position 50). We did not find any *Mnemiopsis *genes with an S at position 50. The only other *Mnemiopsis *genes with a K at position 50 are the 18 SINE class genes that, like the K50 PRD class genes, also characteristically have a K residue at position 50.

Consistent with the absence of lysine or serine residues at position 50 in *Mnemiopsis *and *Amphimedon *PRD homeodomains, we see no grouping of *Mnemiopsis *or *Amphimedon *homeodomains with S50 or K50 clades, with the following exceptions: (1) MlPRD16 groups with the *Nematostella *S50 homeodomain NvPRD074, albeit with virtually no support (ML bootstrap = 2, Bayesian posterior probability distribution = 2), within a larger clade of Q50 homedomains; and (2) the *Amphimedon *homeodomain AqQ50a groups with the highly divergent HsDUXBI (ML bootstrap = 27, Bayesian posterior probability distribution = 95), also within a larger clade of Q50 homedomains (Figure 1). The overwhelming evidence suggests that *Mnemiopsis *and *Amphimedon *are devoid of S50 and K50 PRD class homeodomains. Conversely, *Nematostella *and *Trichoplax *both have clear K50 and S50 homeodomains. The phylogenetic distribution of Q50, S50 and K50 PRD homeodomains in our study is consistent with the hypothesis that Q50 homeodomains were the founders of the PRD class [44].

Five of the seven *Mnemiopsis *PRD class homeodomains contain the diagnostic residues (L[EINQRV][^DGHMPTVWY][^CDGKMNPQR][FL][^CFILPTWY][AEFHKQRV][ADEGKNSTW][CHKMPQR][FHY]P at positions 16-26) associated with paired homeodomains in bilaterians: MlPRD10a, MlPRD10b, MlPRD16, MlPRD44, MlPRD61 (Table 1). No other *Mnemiopsis *homeodomains display this pattern. MlPRD10b, MlPRD16 and MlPRD44 had been identified as Paired class genes in a previous study and were named Prd3, Prd1 and Prd2 respectively [36]. MlPRD44 also contains the HOXL2 diagnostic residues (Table 1).

MlPRD16 and MlPRD61 have clear octapeptide sequences upstream of the homeodomain (SSISSLLS and HSIDDILG, respectively), a hallmark characteristic of a subset of the PRD class homeodomains. MlPRD43 and MlPRD50 have less-conserved but possible octapeptides as well (QRILGILS and YNIEGLLG, respectively). There are no paired domains associated with any *Mnemiopsis *homeodomains, but there are two independent paired domain sequences that appear to be direct orthologs of the two identified in the ctenophore *Coeloplana willeyi *[33].

Like most PRD class homedomains [46], all but one of the *Mnemiopsis *PRD homeodomains have an intron that occurs in the vicinity of the 46th and 47th codons. The one exception, MlPRD10a, has a single intron that interrupts the 37th codon. This might be the result of a retrotransposition event involving a transcript from its paralog (MlPRD10b) followed by an intron gain event. There are additional introns in the N-termini of the homeodomains of MlPRD10b, MlPRD50, and MlPRD61.

### POU class

**Figure 4:**
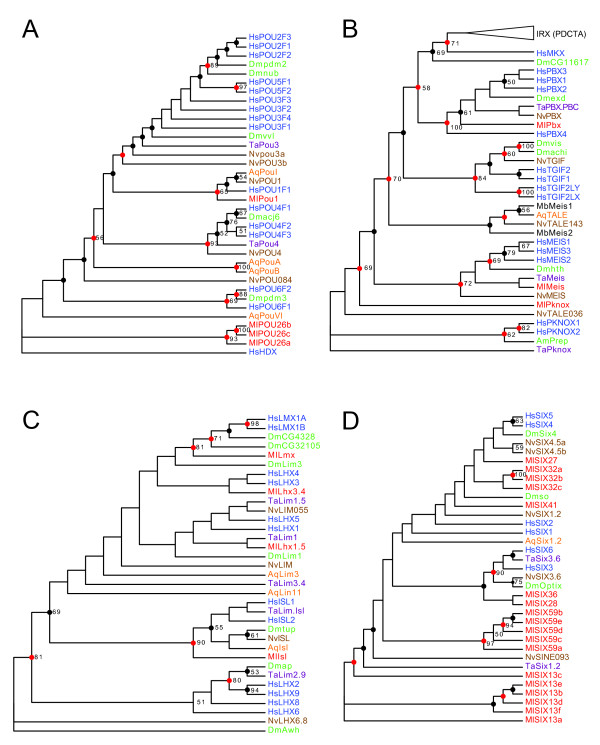
**Trees for other classes**. The remaining class trees are as follows: (A) POU class; (B) TALE class; (C) LIM class; and (D) SIX class. See Figure 2 for an explanation of tree formatting, colour codes and species codes. Branch lengths are presented uniformly for display purposes. Actual branch lengths can be viewed by opening the Newick-formatted tree file (Additional File 4), which also includes bootstrap and Bayesian support values, in a tree viewing/editing program such as FigTree [53].

MlPOU1, MlPOU26a, MlPOU26b, and MlPOU26c make up the four *Mnemiopsis *POU class homeodomains. MlPOU1 has relatively strong support values, placing it in the POU1 family (ML bootstrap = 65; Bayesian posterior probability distribution = 98; Figure 4A). In addition, it has a POU-specific domain upstream of the homeodomain, a defining factor of the POU class [47]. There is weak support uniting MlPOU26a, MlPOU26b and MlPOU26c with the human HDX (highly divergent homeobox) homeodomain of POU class genes (ML bootstrap = 19; Bayesian posterior probability distribution = 45). Only one of the three MlPOU26 homeodomains (MlPOU26a) contains an upstream POU-specific domain.

### TALE class

MlPbx, MlMeis and MlPknox, like other TALE class homeodomains, have a three amino acid insertion in the loop between the first and second alpha-helices (Table 1). MlPbx, MlMeis and MlPknox consistently group with the Pbx, Meis and Pknox families, respectively, in both trees with moderate support (Figures 1 and 4B). In all three cases, the phylogenetic assignment of these homeodomains is reinforced by the identification of several conserved motifs outside of the homeodomain, as well as by conserved intron positions (Table 1).

Like other Pbx genes (and unlike other TALE genes), MlPbx has a glycine residue at position 50 of the homeodomain. In addition, a Basic Local Alignment Search Tool (BLAST) search to the contig containing MlPBX shows significant similarity to the PBC domain [48] located ~1.5 KB upstream of the homeodomain, as assessed by BLAST [percent identity (ID)= 25/67, expectation (E)-value = 2 × 10^-7^). Like the cnidarian and human PBX genes, MlPbx has an intron that interrupts the second codon and one that falls between the 47th and 48th codon of the 63-codon TALE homeobox.

Meis homeodomain proteins have several conserved motifs in addition to the homeodomain [49]. A GENSCAN prediction containing the MlMeis homeodomain shows similarity to the upstream MEIS A domain (ID = 19/69, E-value = 0.005), as well as weaker similarity to the MEIS D domain downstream of the homeodomain (ID = 16/48, E-value = 0.014). Similar to bilaterians and cnidarians, MlMeis has two introns. One falls between the 25th and 26th codons, while another interrupts the 51st codon.

The GENSCAN-predicted peptide that contains the MlPknox homeodomain also includes the abbreviated MEIS A domain that is characteristic of the Pknox family, as well as the MEIS B motif (ID = 33/139, E-value = 8 × 10^-5^). MlPknox, like the human PKNOX1 and PKNOX2 genes, has an intron that separates the 25th and 26th codons and one that interrupts the 51st codon of the homeobox.

We were unable to identify an Irx homeodomain in *Mnemiopsis*, despite there being Irx family members from *Amphimedon, Trichoplax *and *Nematostella*. Also absent was the Tgif homeodomain, found only in cnidarians and bilaterians.

### LIM class

MlIsl, MlLhx1.5, MlLhx3.4 and MlLmx make up the four LIM class homeodomains of *Mnemiopsis *(Figure 4C). We assigned these four *Mnemiopsis *homeodomains to the Isl, Lhx1/5, Lhx3/4 and Lmx families, respectively, based on the consistency between tree runs (Figures 1 and 4C) and moderate support in the full homeodomain tree (Figure 1). BLAST searches of the genomic scaffolds containing *Mnemiopsis *LIM homeodomains reveal LIM-type zinc finger domains immediately upstream of these four homeodomains. Additional BLAST searches also reveal traces of LIM domains independent of homeodomains in the *Mnemiopsis *genome (data not shown), suggesting the existence of LIM domain transcription regulator genes.

### SINE class

Eighteen SINE class homeodomains representing seven distinct SINE lineages were recovered from the *Mnemiopsis *genome (Figure 4D, Table 1). Of these, all but one have the characteristic lysine at position 50 (as described in [21]). The exception is MlSIX41, for which we are missing the sequence information from the C-terminus of the homeodomain (including position 50). Additionally, 17 of the 18 SINE class homeodomains have a SIX domain upstream of the homeodomain. The exception, MlSIX32a, is situated on the N-terminal end of a small scaffold in our current assembly, so its absence may be due to the resolution of our assembly.

The SINE class is monophyletic in our superfamily tree except for a clade of five *Mnemiopsis *homeodomains (MlSIX59a, MlSIX59b, MlSIX59c, MlSIX59 d and MlSIX59e), which group with Zhx/Homez (Figure 1). This exception is perhaps not completely unexpected given that, like the Zhx/Homez genes, MlSIX59 homeodomains are quite divergent; they are five of only six homeodomains in our entire *Mnemiopsis *set that do not include a tryptophan at position 48, which is characteristic of the typical homeodomain. The other homeodomain, MlSIX45, is also a member of the SINE class.

The *Mnemiopsis *SINE class homeodomains do not clearly separate into the three families recognized in bilaterians. Only two of the 18 maintain the four family-defining diagnostic residues (positions 3-6) in the homeodomain [50]. MlSIX41 and MlSIX27 have the SIX1/2 family 'ETSY' pattern in positions 3-6 of the homeodomain. However, neither MlSIX41 nor MlSIX27 group convincingly with the Six1/2 group. The *Mnmemiopsis *SIX class is the result of extensive ctenophore-specific diversification. A more in-depth phylogenetic analysis that includes SIX domains may provide additional insight into these relationships.

### Unclassified *Mnemiopsis *homeodomains

Two clades consisting of 18 *Mnemiopsis *homeodomains appear as separate offshoots in our superfamily tree (Figure 1, Table 1). None of these 18 homeodomains have introns, or any of the known class signatures, that would hint that they might belong to an existing class. MlHD60 and MlHD79 have insertions that interrupt the homeodomain but these insertions are unlike the known insertions seen in the TALE, HNF and PROS classes. The MlHD60 insertion consists of two amino acids that occur in the third alpha-helix. The other insertion occurs in the loop region between the first and second alpha-helices but, unlike the TALE insertions, it consists only of a single amino acid. The average branch length of the homeodomains in these clades is 5% shorter than for the other *Mnemiopsis *homeodomains, confirming that these unclassified Mnemiopsis homeodomains do not simply comprise a clade of unusually long branches.

### Missing classes

There are no *Mnemiopsis *homeodomains that grouped with HNF, CUT, PROS, or CERS classes in our analyses. Consistent with this result, no *Mnemiopsis *homeodomains exhibit insertions between the second and third helices, like those seen in the bilaterian HNF and PROS class homeoboxes. Besides the five apparent SINE class homeodomains, no other *Mnemiopsis *homeodomains group with zinc finger (ZF) homeodomains.

### Homeobox linkage

**Figure 5:**
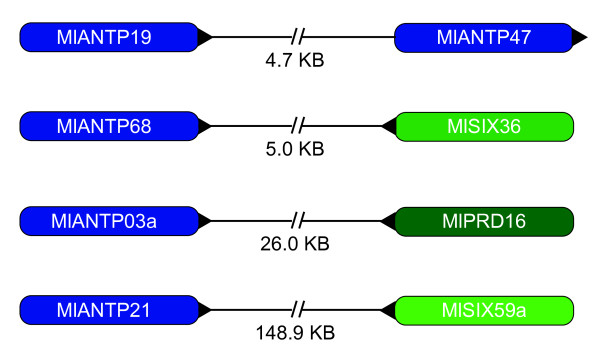
**Homeobox linkage in *Mnemiopsis***. These four pairs of homeoboxes are linked on four pairs of genomic scaffold. The distance between homeoboxes is noted in between each pair. Arrowheads applied to one side or the other represent the orientation of the linkage.

There are four pairs of linked homeoboxes in our current *Mnemiopsis *genome assembly (Figure 5). The tightest linkage is between two ANTP class homeoboxes (MlANTP19 and MlANTP47), which are 4.7 KB apart. A different ANTP class homeobox (MlANTP68) is situated 5.0 KB downstream from the SINE class homeobox MLSIX36. The HOXL-related ANTP class homeobox MlANTP03a is separated by 26.0 KB from the PRD class homeobox MLPRD16. The ANTP class MlANTP21 and the SINE class homeobox MLSIX59 are on the same contig, 148.9 KB apart. None of the linked homeoboxes are obvious paralogs, suggesting that these pairs are not the result of recent duplication events.

### Evolutionary dynamics of the *Mnemiopsis *homeodomain superfamily

In order to better-understand the nature of the homeodomain superfamily in *Mnemiopsis*, we compared average branch lengths and the number of species-specific homeodomain clades in the *Mnemiopsis*, *Amphimedon*, *Trichoplax*, *Nematostella*, *Drosophila*, *Caenorhabditis elegan*, and human genomes (Table 2). We performed ML analyses with homeodomain sequences from this set of species. Degenerate homeodomains (for example, *Amphimedon *PaxB) and homeodomains from pseudogenes were not included. The resulting tree and alignments are included as supplemental material (Additional file 2).

**Table 2:** Paralog count and estimated branch lengths of seven species.

Species	No. of HDs in this test	No. of HDs in species-specific clades	No. of species-specific clades	Average branch length
*Nematostella vectensis*	127	66	22	1.010
*Drosophila melanogaster*	102	25	12	1.052
*Trichoplax adhaerens*	35	0	0	1.162
*Amphimedon queenslandica*	31	7	2	1.286
*Homo sapiens*	256	197	74	1.293
*Mnemiopsis leidyi*	76	45	14	1.344
*Caenorhabditis elegans*	113	34	11	1.480

**Average**	105.71	53.43	19.29	1.232

The second column specifies the number of homeodomains (HDs) used in the neighbor-joining analyses that produced the tree from which this table is based. The third column indicates the number of homeodomains that were more closely related to a homeodomain from the same species. The number of species-specific clades is denoted in the fourth column. The final column shows the average length of all branches from a particular species based on a midpoint rooted tree. Files used in this analysis are (Additional File 2).

For each species, we recorded the number of species-specific clades that included more than one homeodomain, as well as the total number of homeodomains in those species-specific clades (Table 2). These numbers give us an approximation of the number of lineage-specific homeodomain duplications that have been preserved in a specific lineage since it split from its closest relative in the analysis [51]. A species that has recently undergone extensive genome reduction would be expected to harbour less species-specific clades than a genome that has experienced a recent genomic expansion. Our data shows that very few paralogous homeodomains exist in the *Amphimedon *(7) and *Trichoplax *(0) genomes, whereas the human genome has a remarkably high level of paralogs (197). *Mnemiopsis *(45) and *Nematostella *(66) are both very close to the mean (Table 2).

Branch lengths provide a means of measuring the level of divergence for a particular homeodomain. Longer branches correspond to higher levels of divergence. We rooted the same neighbor-joining tree described above at its midpoint and determined the average branch lengths for each species' set of homeodomains (Table 2). In our tree, the *Mnemiopsis *branches tend to be longer than for all the other species except for *C. elegans*, which is known to have very long branches [52]. The *Mnemiopsis *average branch length is slightly closer to the mean than it is to the *C. elegans *average, suggesting that the *Mnemiopsis *homeodomains are moderately divergent. The trees used in this analysis are included as supplemental material (Additional Files 2 and 3) and branch lengths can be visualized directly using a tree-viewing program such as Figtree [53].

## Discussion

**Figure 6:**
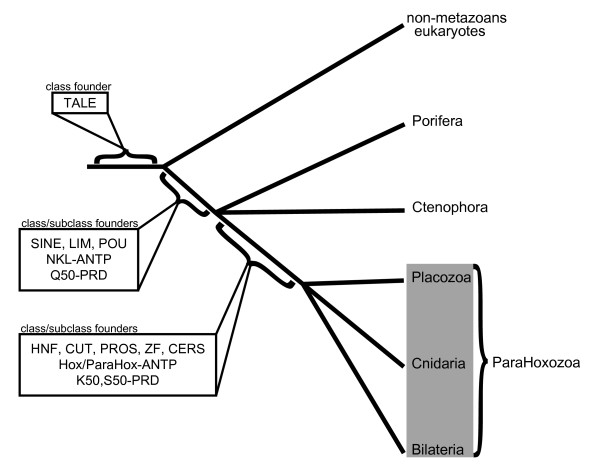
**Phylogenetic relationship of early-branching animal lineages**. Given the conflicting results of several recent studies and the current existing phylogenomic evidence presented in this study, we have constructed an animal phylogeny that includes two polytomies. It is uncertain whether Porifera or Ctenophora branched at the base. The evidence from this study (and others) unites Placozoa, Cnidaria and Bilateria into the subkingdom ParaHoxozoa. The origins of class and subclass founders are indicated along major branches of the tree.

There is strong evidence suggesting that *Mnemiopsis *has homeodomains belonging to six of the 11 defined homeodomain classes (ANTP, PRD, LIM, POU, SINE and TALE). *Mnemiopsis *appears to be missing the other five homeodomain classes (HNF, CUT, PROS, ZF and CERS). It is also missing Â¬the Hox/ParaHox and extended Hox subclasses of the ANTP class, as well as the S50 and K50 subclass of the PRD class. Given that *Trichoplax*, *Nematostella *and the bilaterians examined in our study clearly possess all of these classes and subclasses, the most parsimonious animal tree would involve Ctenophora and Porifera branching off the main animal trunk *prior *to the Placozoa, Cnidaria and Bilateria (Figure 6). This configuration is congruent with most of the previously published 18 S phylogenies and results from several of the EST-based phylogenomic studies [12,15].

### ParaHoxozoa

The apparent absence of Hox/ParaHox, S50 and K50 PRD and HNF class homeodomains in *Amphimedon *and *Mnemiopiopsis *supports an organismal clade that unites cnidarians, bilaterians and placozoans. Although previous analyses have strongly suggested a monophyletic relationship of these three phyla (particularly Collins [6]), we are not aware of any studies to date that have given this subkingdom a name. We propose the name ParaHoxozoa for the clade containing Bilateria, Placozoa and Cnidaria.

Studies have shown that the ancestor of the ParaHoxozoa had at least one Hox/ParaHox gene [26,54] and that these genes are missing from both ctenophores and sponges ([55] and this study). While cnidarians and bilaterians have homeodomains that are clearly descended from both ancestral Hox and ParaHox genes, *Trichoplax*, has only a single Hox/ParaHox gene, Trox2 [54]. This homeodomain consistently forms a moderately supported clade with the ParaHox GSX family ([28,56] and this study) and may be a true ParaHox gene. However, it has been postulated that the Trox2 gene may be a direct descendant of an ancestral 'ProtoHox' gene rather than a proper ParaHox gene [28,54,56]. The name ParaHoxozoa was chosen since, based strictly on the trees themselves, a ParaHox clearly unites this group.

### Evidence distinguishing *Mnemiopsis *from the ParaHoxozoa

The presence of ample paralogs in the *Mnemiopsis *genome suggests that it has not undergone extensive genome reduction. Currently, we cannot rule out the possibility that recent duplication events have masked more ancient gene losses or that the paralog count in the homeobox superfamily is not typical of most superfamilies. Regardless, the level of paralog retention does give some initial insight into the evolutionary dynamics of the *Mnemiopsis *genome, while also lowering the probability that the *Mnemiopsis *lineage at one time had (and subsequently lost) these missing homeodomains. The availability of other ctenophore genomes for future studies and analysis of other gene superfamilies will help to better-resolve the evolutionary dynamics of the *Mnemiopsis *genome.

The unusually high level of conservation of the homeobox genes and their vital roles in early development further lowers the possibility that the loss of entire classes and subclasses of these genes could be tolerated. There are numerous examples of homeobox families missing from a wide array of phylogenetically disparate lineages. For example, *Ciona intestinalis *and multiple species of parasitic platyhelminthes appear to have lost several Hox genes [57,58]. In addition, the genomes of *Drosophila *and human are each missing several homeobox families [26]. However, it is important to note that no species examined thus far has been shown to be missing entire classes or subclasses of homeobox genes.

Long branches do have the ability to distort phylogenies and the presence of longer branches in our *Mnemiopsis *data set was initially a concern. However, if ctenophores are among the earliest metazoan branches and substantial expansion of the homeodomain superfamily occurred along the lineage leading to *Mnemiopsis*, these long branches are not entirely unexpected. Conversely, the somewhat shorter branches in *Amphimedon *homeodomains, despite its early-branching phylogenetic position, are likely due to it having far fewer paralogs.

Another possible concern is that our paralog results are an artifact caused by the attraction of long branches and that these long branches represent class members that appear to be missing but are perhaps unrecognizable in *Mnemiopsis*. Two pieces of evidence undermine this assertion. First, *C. elegans*, which has 9% longer branches than *Mnemiopsis *and 67% more homeodomains, would be expected to have more species-specific homeodomain clades, but in fact has 23% fewer (Table 2). Second, when we remove the *C. elegans *homeodomains from the dataset (which vastly reduces the number of long branches in our tree) and redo the analysis, the number of *Mnemiopsis*-specific clades does not change (Additional File 3).

### Incongruence with the 'Coelenterate' hypothesis

A scenario grouping cnidarians and ctenophores into a 'coelenterate' clade consistent with the phylogenomic study by Philippe *et al*. [10] seems unlikely based on the number of clades that include a *Nematostella *homeodomain to the exclusion of a *Mnemiopsis *representative. For example, *Nematostella *has representatives of the ANTP homeodomain families Mox, Emx, Gbx, Ro, Mnx, Vax, Not, Nk1, NK2 and NK3, as well as two ParaHox and three Hox-related homeoboxes (Figure 2). If Ctenophora and Cnidaria were, in fact, sister taxa, an extraordinary number of gene losses would have been required in the *Mnemiopsis *lineage, given the observation that *Mnemiopsis *is lacking these families. This amount of loss seems very unlikely given the pattern of paralog retention in *Mnemiopsis*. A more recent study by this group also failed to recover the 'coelenterate' clade [15].

### *Mnemiopsis *extended Hox?

The origin of the four paralogous *Mnemiopsis *ANTP homeodomains (MlANTP03a, MlANTP03b, MlANTP03c and MlANTP03d) that group with the engrailed family in the superfamily tree (Figure 1) and with the Evx family in the ANTP tree is difficult to interpret. The presence of the HOXL signature in two of these four homeodomains, combined with their tendency to form a clade with hox-related genes, suggests that these four homeodomains may have descended from an ancestral homeodomain; this ancestral homeodomain may then, in turn, have led to the formation of the extended Hox (Evx, Gbx, Meox, Mnx, Rough), Hox and ParaHox classes through a series of duplications in the ParaHoxozoa stem.

Functional analysis of the MlANTP03 genes may give further insight into the role of this ancestral gene, which would be particularly interesting given the roles that extended Hox genes play in critical biological processes such as neurogenesis, myogenesis, axial patterning, segmentation, gastrulation and photoreception.

### Identifying the basal branch

Despite strong evidence uniting the ParaHoxozoa to the exclusion of Porifera and Ctenophora, it does not appear that homeodomain sequences alone (at least based on the current set of available sequences) will be powerful enough to incontrovertibly determine the basal metazoan branch. There are several unambiguous clades containing *Mnemiopsis *homeodomains grouping with parahoxozoan clades to the exclusion of *Amphimedon *representatives (for example, Lmx, Hlx and Pbx). The many occurrences of this pattern insinuate that sponges branched off at the base of the Metazoa. This pattern is consistent with many of the 18 S phylogenies (see Table 1 in [59] and references therein), as well as a recent EST-based phylogeny [15]. However, there is substantial evidence suggesting the alternative relationship.

A number of parahoxozoan clades include *Amphimedon *homeodomains but lack a corresponding *Mnemiopsis *homeodomain (for example, NK2 and Irx). The scarcity of paralogous homeodomains could be a sign that the *Amphimedon *genome is reduced. This, in turn, increases the chance that multiple homeodomain families were lost in *Amphimedon*. In addition, the lack of a homeobox associated with a paired domain in *Mnemiopsis *opens the possibility that the fusion of the homeobox and paired domain postdates the divergence of ctenophores from the rest of Metazoa. This would be consistent with other phylogenomic studies [11,12].

It is possible that similar analyses of additional gene families with the same set of taxa might be able to resolve the basal branch. Alternatively, it could be that sequence data from additional sponge and ctenophore genomes will be required to satisfactorily settle the debate. The current study does make a strong case that this uncertainty might eventually be resolved using a gene family-type approach.

### Implications for the evolution of homeobox clusters

Extensive genomic clustering of ANTP homeoboxes in multiple metazoan genomes suggests that the ANTP class homeobox genes were formed by a series of tandem duplications (reviewed in [60]). A recent study showed that six of the eight *Amphimedon *NKL homeoboxes are clustered [30] and are likely descendants of the ancestral ANTP megacluster. One of the four *Mnemiopsis *homeobox clusters - specifically, the cluster containing MlANTP19 and MlANTP47 - is potentially a remnant of this ANTP megacluster as well (Figure 5). In our phylogenetic analyses, MlANTP47 consistently groups with the *Amphimedon *NK5.7b homeodomain with moderate support (ML bootstrap = 24, Bayesian posterior probability distribution = 95; Figure 2). The family-level identity of MlANTP19 is uncertain but it does not appear to be paralogous with MlANTP47.

It is very difficult to draw any conclusions as to the implications of the other three linked homeoboxes, particularly given the difficulty of assigning family-level orthology to the *Mnemiopsis *homeodomains. The process for detecting ancestral/functional linkages involves identifying orthologs in multiple evolutionarily disparate genomes. Therefore, it may be possible that these linkages are significant and, perhaps, representative of some ancestral cluster, but the current phylogenetic resolution of the *Mnemiopsis *homeoboxes may make this relationship difficult to detect.

## Conclusions

We have identified, named and classified 76 homeoboxes in the *Mnemiopsis leidyi *genome. In many cases, we have provided additional evidence for our classifications through the detection of diagnostic residues, presence of surrounding domains and identification of conserved intron positions. We have shown that several classes and subclasses are present in placozoan, cnidarian and bilaterian species but are missing from both *Mnemiopsis *and the sponge *Amphimedon *(Table 3). Using a phylogenomic approach, we have determined that it is very likely, based on the presence and absence of homeodomains, that the phyla Placozoa, Cnidaria and Bilateria are more closely related to each other than they are to Ctenophora or Porifera. Based on this evidence, we have proposed the name ParaHoxozoa for the clade that includes Placozoa, Cnidaria and Bilateria.

**Table 3:** Distribution of classes and subclasses of homeobox genes among early branching taxa.

	Ancestral eukaryote	*Amphimedon*	*Mnemiopsis*	*Nematostella*	*Trichoplax*	Human/*Drosophila*
**ANTP**						

Hox	**--**	**--**	**--**	**+**	**+**	**+**

ParaHox	**--**	**--**	**--**	**+**	**+**	**+**

Extended Hox	**--**	**--**	**+**	**+**	**+**	**+**

NKL	**--**	**+**	**+**	**+**	**+**	**+**

**PRD**						

Q50	**--**	**+**	**+**	**+**	**+**	**+**

N50	**--**	**--**	**--**	**+**	**+**	**+**

K50	**--**	**--**	**--**	**+**	**+**	**+**

**LIM**	**--**	**+**	**+**	**+**	**+**	**+**

**POU**	**--**	**+**	**+**	**+**	**+**	**+**

**SINE**	**--**	**+**	**+**	**+**	**+**	**+**

**TALE**	**+**	**+**	**+**	**+**	**+**	**+**

**HNF**	**--**	**--**	**--**	**+**	**+**	**+**

**CUT**	**--**	**--**	**--**	**--**	**--**	**+**

**PROS**	**--**	**--**	**--**	**--**	**--**	**+**

**ZF**	**--**	**--**	**--**	**--**	**--**	**+**

**CERS**	**--**	**--**	**--**	**--**	**--**	**+**

The first column contains the class (bold) or subclass (indented). A plus indicates the presence of a clear member of the class or subclass. Alternatively, a minus indicates the absence of a clear member of the class or subclass. Inference of the homeodomain complement of the ancestral eukaryote is based on Derelle *et al*. [22].

The expansion of the homeobox superfamily has played a major role in the evolution of animal phyla [61]. An understanding of this expansion, along with an accurate animal phylogeny, is critical to understanding metazoan evolution. With this new dataset and phylogeny, as well as the help of functional genomic techniques, we can start piecing together the evolutionary steps that led to such astounding evolutionary feats such as the development of nervous systems, muscular systems, and complex symmetry.

## Methods

### Sequencing and assembly

Genomic DNA was isolated from the larvae of two separate self-fertilizing hermaphroditic individuals. A library from one source was sequenced to 10× coverage using 454 sequencing: 8.1 million reads totaling 2.7 gigabases were assembled into 29,877 contigs (contig-N50 = 11KB) using the Phusion assembler [62]. Subsequently, we constructed and sequenced a paired-end DNA library with insert sizes of ~4kb from the second genomic source using Illumina sequencing. These 2.8 million paired end reads were used to compile the contigs into 10,106 scaffolds (scaffold-N50 = 123 KB) bringing the physical coverage to ~50×.

### Retrieval of *Mnemiopsis *homeodomains

A TBLASTN search of the *Mnemiopsis *assembly was conducted using a set of bilaterian homeodomains downloaded from the Homeodomain Resource [63]. This set was compared to and supplemented by previous *Mnemiopsis *homeodomains that were generated from degenerate PCR and RACE [36].

### Gene isolation via RACE PCR

MlPRD86, MlPbx, MlSIX59b, MlSIX41, MlSIX13f, MlPRD10b, MlANTP65, MlANTP67, MlPRD76b, MlPRD60, MlPRD77b and MlPRD86 homeoboxes had been isolated but not published prior to the sequencing of the genome and are included in this study. These were isolated as previously described [36].

### Superclass alignment

The *Mnemiopsis *homeodomains were aligned by eye to the dataset used by Holland *et al*. 2007, which consisted of all human homeodomains and a representative set of protostome set consisting mostly of homeodomains from *Drosophila melanogaster *[23]. We supplemented these sequences with eight *Branchiostoma floridae *homeodomains that are known to be missing from humans. Insertions in the loop-region of the *Mnemiopsis *homeodomains were removed as done in [23] and other studies. In determining which amino acids to remove from atypical homeodomains, we realized that there was inconsistency in the Holland set as to which three amino acids were removed between the *Drosophila *and the human sequences. As with the human sequences, we removed the 23rd, 24th and 25th amino acids from the *Mnemiopsis *atypical TALE sequences and adjusted the *Drosophila *sequences so they conformed to this rule. Alignment is available as supplementary material (Additional File 1).

### Class alignment

The superclass alignment was divided into six separate alignments, (1) ANTP, (2) PRD, (3) SIX, (4) LIM, (5) POU and (6) TALE based on the best superclass tree and the class membership of the bilaterian homeodomains determined from HomeoDB [24]. The three amino acid insertions were reinserted into the TALE alignment. To each of these datasets we added homeodomains from other non-bilaterian species based on published classifications. We added 32 homeodomains from the demosponge *Amphimedon queenslandica *[30], 37 homeodomains from the placozoan *Trichoplax adhaerens *[27], 127 homedomains from the cnidarian *Nematostella vectensis *[26] and two homeodomains from the choanoflagellate *Monosiga brevis *[39]. These alignments are available as supplemental material (Additional File 1).

### Phylogenetic analyses

The Perl script proteinModelSelection.pl (available from the RaxML [37] web site) was used to determine the best scoring amino acid substitution model for our supertree alignment (RTREV +GAMMA). All subsequent analyses used this model.

Three independent runs of RaxML version 7.0.4 [37] were conducted. Two runs used random starting trees with the following command line (raxmlHPC-MPI -m PROTGAMMARTREV -s ALN.phy -#10 -n NAME -k). One run used a neighbor joining starting tree that was generated with default parameters in Phylip version 3.6a3 [64]. The command line for this run was (raxmlHPC -m PROTGAMMARTREV -s ALN.phy -t NJ.tre -n NAME -k).

Three independent runs of PhyML version 3.0 [38] were conducted. Two runs used random starting with the following command line (phyml -i ALN.phy -d aa -m RtRev -a e -q --rand_start -s SPR --r_seed 'cat FILE_W_RANDSEED'). One run used a neighbor joining starting tree with the following command line (beorun phyml -i ALN.phy -d aa -m RtRev -a e -q -s SPR).

Two independent runs with the MPI version of Mr. Bayes version 3.1.2 [65] were conducted with the following execution block (prset aamodelpr = fixed(RTREV); lset rates = gamma; mcmcp mcmcdiagn = no nruns = 1 ngen = 5000000 printfreq = 5000 samplefreq = 500 nchains = 5 savebrlens = yes; mcmc; sumt filename = tale_w_insert.nex nRuns = 1 Relburnin = YES BurninFrac = .25 Contype = Allcompat;). Log likelihood values were plotted and their progression was visually examined over time. All runs were found to be asymptotic before the .25 burnin fraction.

Likelihood values for all runs (3 RaxML, 3 PhyML, 2 Bayes and 1 neighbor joining) were generated using PhyMl version 3.0 [38] with the following command line (phyml -i ALN.phy -c 4 -m RtREV -a e -o lr -f d -u TREE.tre -d aa -b 0 -s NNI). The tree with the highest likelihood value was chosen for all downstream analysis. The best trees were as follows: Supertree = RaxML-randomstart, ANTP = RaxML-randomstart, PRD = RaxML-randomstart, SIX = PhyML-randomstart, LIM = RaxML-randomstart, POU = RaxML-NJstart, and TALE = RaxML-randomstart. The best trees are available as supplemental material (Additional File 4).

### Support indices

Support was assessed by 100 replicates of the bootstrap using the method that provided the highest likelihood value. Bootstrap values greater than 50 were applied to the best tree. To these three trees, Bayesian posterior probabilities were also added from the Bayesian tree with the highest likelihood score. The best trees with support values are available as supplemental material (Additional File 4).

### Paralog retention analysis

From our initial superfamily alignment, we removed all sequences that were not *Mnemiopsis*, *Drosophila*, or human. To this, we added all *Trichoplax*, *Nematostella *and *Amphimedon *sequences that were used in our class-level phylogenies. Finally we added all the *C. elegans *homeodomains that were used in [66]. We ran a neighbor-joining analysis using default parameters in Phylip [64]. This tree was then parsed with a Perl script (count_species_specific_clades.pl) that identified occurrences of homogeneous clades of taxa with identical two-letter prefixes. This Perl script, tree and alignment are included as supplemental material (Additional File 2). A version of this analysis was run without the *C. elegans *data (see Additional File 3).

### Branch length analysis

The tree used in the paralog analysis was subsequently used to estimate average branch lengths (Table 2). This tree was opened in FigTree v.1.2.3 [53], rooted at the midpoint, and saved. This rooted tree was then opened in TreeStat v.1.2 [67] and the Root-Tip Lengths were calculated. A Perl script (calculate_average_root_tip.pl) was used to parse the output of TreeStat and calculate the average branch lengths. This Perl script, tree, and alignment are included as supplemental material (Additional File 2). In order to compare lengths of subsets of *Mnemiopsis *data, we ran a separate instance of this analysis, substituting the two-letter 'Ml' prefix of the subset to be analysed with 'Zz'. In order to assess the feasibility of using this technique to assess the relative branch lengths from trees based on 60 amino acid matrices, we performed a simulation study, and the details of this simulation study are available as supplemental material (Additional File 5).

## Abbreviations

BLAST: Basic Local Alignment Search Tool; E-value: expectation value; EST: expressed sequence tag; HomeoDB: Homeo Database; ID: percent identity; ML: maximum likelihood; PCR: polymerase chain reaction; PHYML: phylogenetic inferences using ML; rRNA: ribosomal RNA; RAxML: randomized accelerated ML; ZF: zinc finger.

## Competing interests

The authors declare that they have no competing interests.

## Authors' contributions

JFR designed and conceived the study, isolated *Mnemiopsis *homeodomains from the genomic assembly, performed alignment, performed phylogenetic analyses and drafted the manuscript. KP isolated *Mnemiopsis *DNA and RNA for sequencing and performed RACE PCR. NISC performed sequencing. JCM assembled *Mnemiopsis *genome. MQM participated in the design of the study and helped to draft the manuscript. ADB participated in the design and conception of the study and helped to draft the manuscript. All authors read and approved the final manuscript.

## Supplementary Material

Additional file 1 **Zip file of alignments**. Includes all alignments used for the supertree and class trees in Phylip format. Click here for file

Additional file 2 **Zip file with data from paralog and branch length analysis**. Includes files necessary to generate Table 2. See 00-README in the zip file for additional information. Click here for file

Additional file 3 **Paralog count and estimated branch lengths of all species in **Table 2** plus *C. elegans***. Includes a table similar to Table 2 and the necessary files to create this table. See 00-README in the zip file for additional information. Click here for file

Additional file 4 **Zip file of trees**. Includes the trees with the highest likelihood generated for the supertree and the class trees in Newick format. Trees include support values from MrBayes and maximum likelihood bootstraps. See 00-README in the zip file for additional information. Click here for file

Additional file 5 **Simulation analysis to assess the feasibility of estimating relative branch lengths from 60 amino acids**. Includes data used to determine the feasibility of the technique used to assess relative branch lengths from trees based on 60-amino acid matrices (as described in the main text). Click here for file
