# Supplementary material for: The homeodomain complement of the ctenophore Mnemiopsis leidyi suggests that Ctenophora and Porifera diverged prior to the ParaHoxozoa
Source: EvoDevo. 2010 Oct 4;1:9. doi: 10.1186/2041-9139-1-9 (PMC2959044; doi:10.1186/2041-9139-1-9)
Supplement: Additional file 5 — Simulation analysis to assess the feasibility of estimating relative branch lengths from 60 amino acids. Includes data used to determine the feasibility of the technique used to assess relative branch lengths from trees based on 60-amino acid matrices (as described in the main text). [file 2041-9139-1-9-S5.ZIP › AF05_Ryan_et_al_2010.brlen_sim/AF05_Ryan_et_al_2010.brlen_sim.pdf]

ADDITIONAL FILE 5:

We ran a simulation study to address concerns whether there was sufficient signal to correctly infer branch length from so few positions. We created a balanced 16 taxa tree (see below) and used Seq-Gen (v1.3.2) to create 100 simulated datasets.

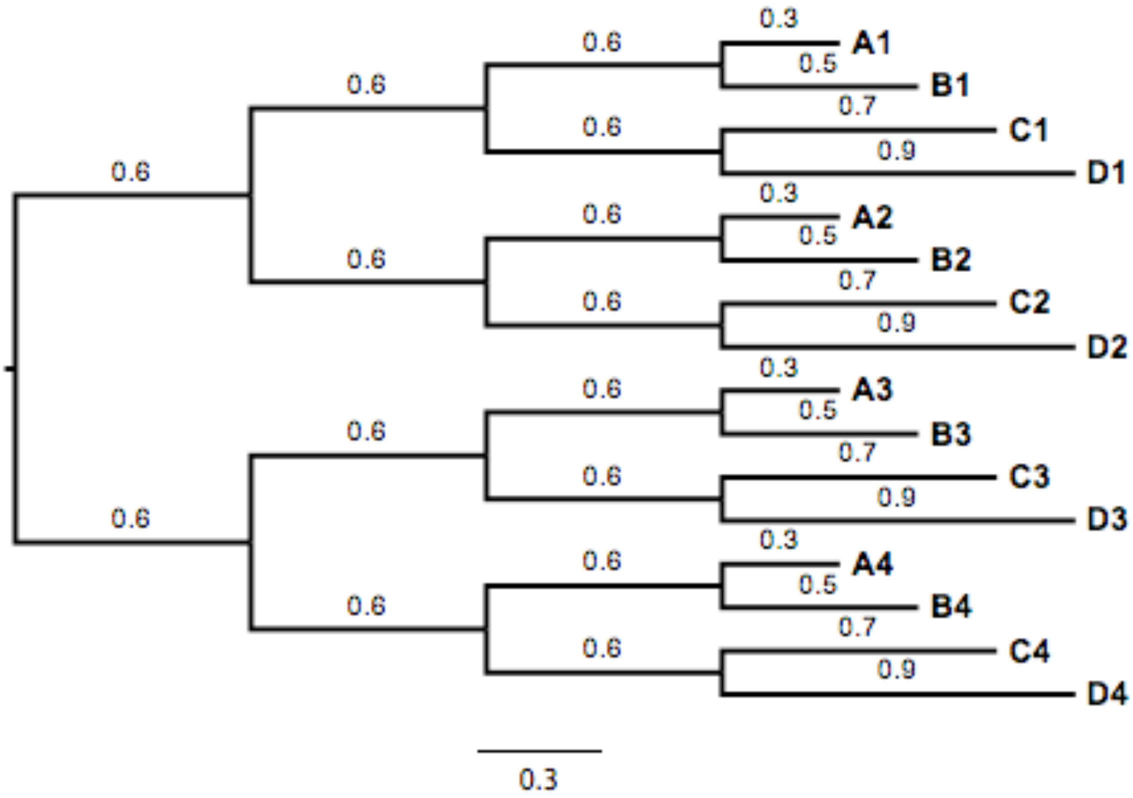

Tree used to simulate 100 datasets. Branch lengths are indicated above each branch. The one-letter prefixes of each taxa represent individual species. Taxa with an A prefix have root-tip branch lengths of 2.1. Taxa with a B prefix have root-tip branch lengths of 2.3

To estimate the branch lengths for our simulation tree, we rooted our seven-species tree (Additional File 3) at the TALE clade and measured the root-tip lengths of this rooted tree using TreeStat. We then averaged all the root-tip lengths (2.48). We set the length of the internal branches of our 16-taxa tree to each at 0.6 and set the tip branches of four of the taxa (A1,A2,A3,A4) to be 0.3; four of the taxa (B1,B2,B3,B4) to be 0.5; four of the taxa (C1,C2,C3,C4) to be 0.7; and four of the taxa (D1,D2,D3,D4) to be 0.9 long. This gives our simulated tree an average branch length of 2.4.

We simulated 100 amino acid datasets using this tree and the *Drosophila* ANTP sequence as the ancestral sequence for all simulations with the following command:

```
seq-gen -mJTT -g 4 -q -k1 -z$rand < antp.phy > gen.phy
```

We performed a maximum likelihood analysis on each dataset with the following command:

```
raxmlHPC-MPI -m PROTGAMMAJTT -#10 -n gen -k -s gen.phy
```

We then performed the same branch length analysis we did in our paper on each of these trees (i.e., root at the midpoint and then calculate average branch lengths for the A taxa, the B taxa, C taxa, and D taxa). We limited our simulation to 100 simulations because our branch length analysis requires two manual steps that make processing simulations take a long time.

We plotted the average branch lengths from each of the four taxa (i.e., A,B,C,and D) in R with a box and whisper plot (see below). As expected, the mean of A is less than the mean of B, B is less than the mean of C, and C is less than the mean of D. We performed a T-test comparing all taxa averages against all other taxa averages (i.e., A vs. B, B vs. C, C vs. D, A vs. C, A vs. D, and B vs. D). All T-tests produced significant P-values:

```
t.test(A,B) p-value = 0.00832
t.test(A,C) p-value = 1.811e-06
t.test(A,D) p-value = 7.094e-12
t.test(B,C) p-value = 0.02290
t.test(B,D) p-value = 2.378e-06
t.test(C,D) p-value = 0.007858
```

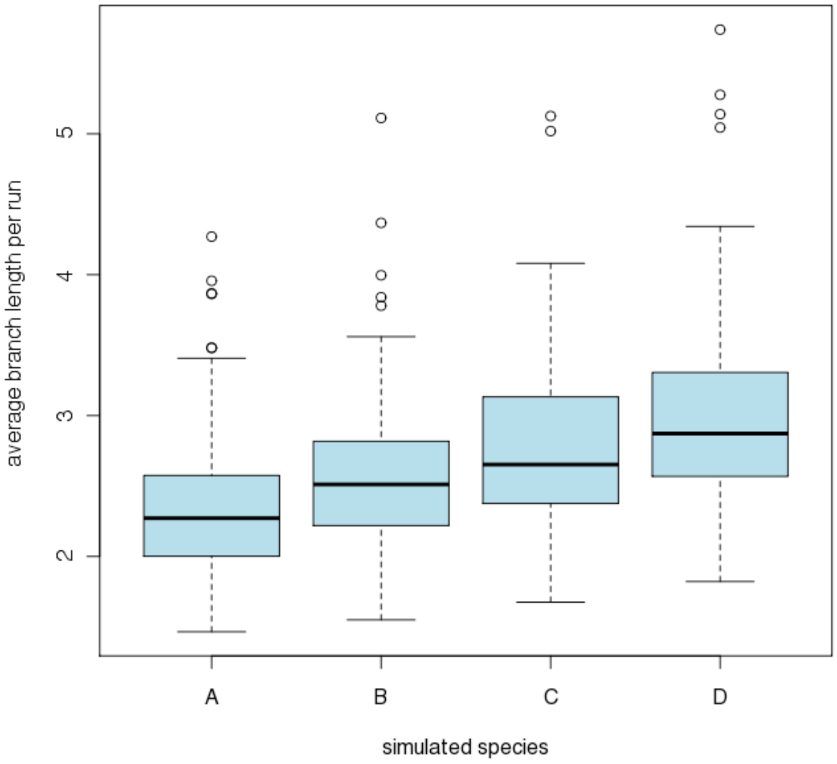

Box and whisker plot of average branch lengths. Thick black line shows the median. This line intersects the blue boxes, which represent the quartiles. The dotted lines extend to the minimum and maximum values except for the open circles, which represent values R by default labeled as outliers.

This simulation addresses the concerns that there is sufficient signal to correctly infer branch length from 60 amino acids. There is clearly branch length signal in this data.
